# GRASPing the unconventional secretory machinery to bridge cellular stress signaling to the extracellular proteome

**DOI:** 10.15698/cst2021.11.259

**Published:** 2021-10-15

**Authors:** Constantinos Demetriades, Julian Nüchel, Markus Plomann

**Affiliations:** 1 Max Planck Institute for Biology of Ageing (MPI-AGE), 50931 Cologne, Germany.; 2 University of Cologne, Cologne Excellence Cluster on Cellular Stress Responses in Aging-Associated Diseases (CECAD), 50931 Cologne, Germany.; 3 University of Cologne, Faculty of Medicine and University Hospital Cologne, Center for Biochemistry, 50931 Cologne, Germany.

**Keywords:** mTORC1, unconventional protein secretion (UPS), GRASP55, Tuberous Sclerosis Complex (TSC), cellular stress response, Golgi, rapamycin

## Abstract

Cellular adaptation to stress is a crucial homeostatic process for survival, metabolism, physiology, and disease. Cells respond to stress stimuli (e.g., nutrient starvation, growth factor deprivation, hypoxia, low energy, etc.) by changing the activity of signaling pathways, and interact with their environment by qualitatively and quantitatively modifying their intracellular, surface, and extracellular proteomes. How this delicate communication takes place is a hot topic in cell biological research, and has important implications for human disease.

Part of the cellular response to stress is an evolutionarily conserved, poorly-understood process called unconventional protein secretion (UPS). Although cells shut down conventional/bulk secretion to save resources upon stress, UPS is induced by specific stress stimuli, and serves as an alternative delivery route by which selected cargo proteins are transported to the plasma membrane or into the extracellular space. At the molecular level, we and others had previously shown that GRASP55, a Golgi-residing protein, facilitates unconventional secretion of distinct proteins. The identity of the very few proteins that were shown to be secreted via UPS highlighted it as a crucial physiological process with potential links to human disease (e.g., cystic fibrosis) and therapeutics. However, despite the emerging importance of UPS for cellular adaptation to stress, several key aspects of its regulation and its biological role remained very poorly understood.

mTOR complex 1 (mTORC1) is a key player in cellular signaling, and dysregulation of its activity is commonly associated with various diseases and the ageing process. In our recent study (Nüchel et al., 2021), we identified UPS as a cellular function that is regulated by mTORC1, and showed that GRASP55 is a novel mTORC1 substrate, thus establishing GRASP55 as the first Golgi-based effector of mTORC1 and revealing a physiological role for mTORC1 at the Golgi. Using GRASP55 as a proxy for UPS activation, we revealed the cell biology of its regulation. In non-stressed cells, active mTORC1 phosphorylates directly GRASP55 to retain it at the Golgi, hence blocking UPS. In contrast, mTORC1 inhibition (by nutritional, pharmacological, genetic means; or in response to any mTORC1-inhibiting stress), leads to GRASP55 dephosphorylation and its relocalization to secretory vesicles, such as autophagosomes and multivesicular bodies (MVBs), to facilitate UPS (**Fig. 1**). Of note, by artificially tethering GRASP55 at the Golgi surface, we also showed that GRASP55 relocalization is a prerequisite for UPS induction upon stress. Furthermore, a non-phosphorylatable GRASP55 mutant already colocalized with autophagosomes and MVBs even in cells with active mTORC1, and showed enhanced secretion of a key UPS cargo protein (i.e., MMP2; discussed below), further highlighting the importance of GRASP55 phosphorylation for UPS induction. Of note, UPS is dysregulated in cells with aberrant mTORC1 activity (e.g., in a cellular model of Tuberous Sclerosis), thus raising the plausible hypothesis that it may be contributing to the pathology of various mTOR-opathies, and setting the ground for future studies in this direction.

**Figure 1:**
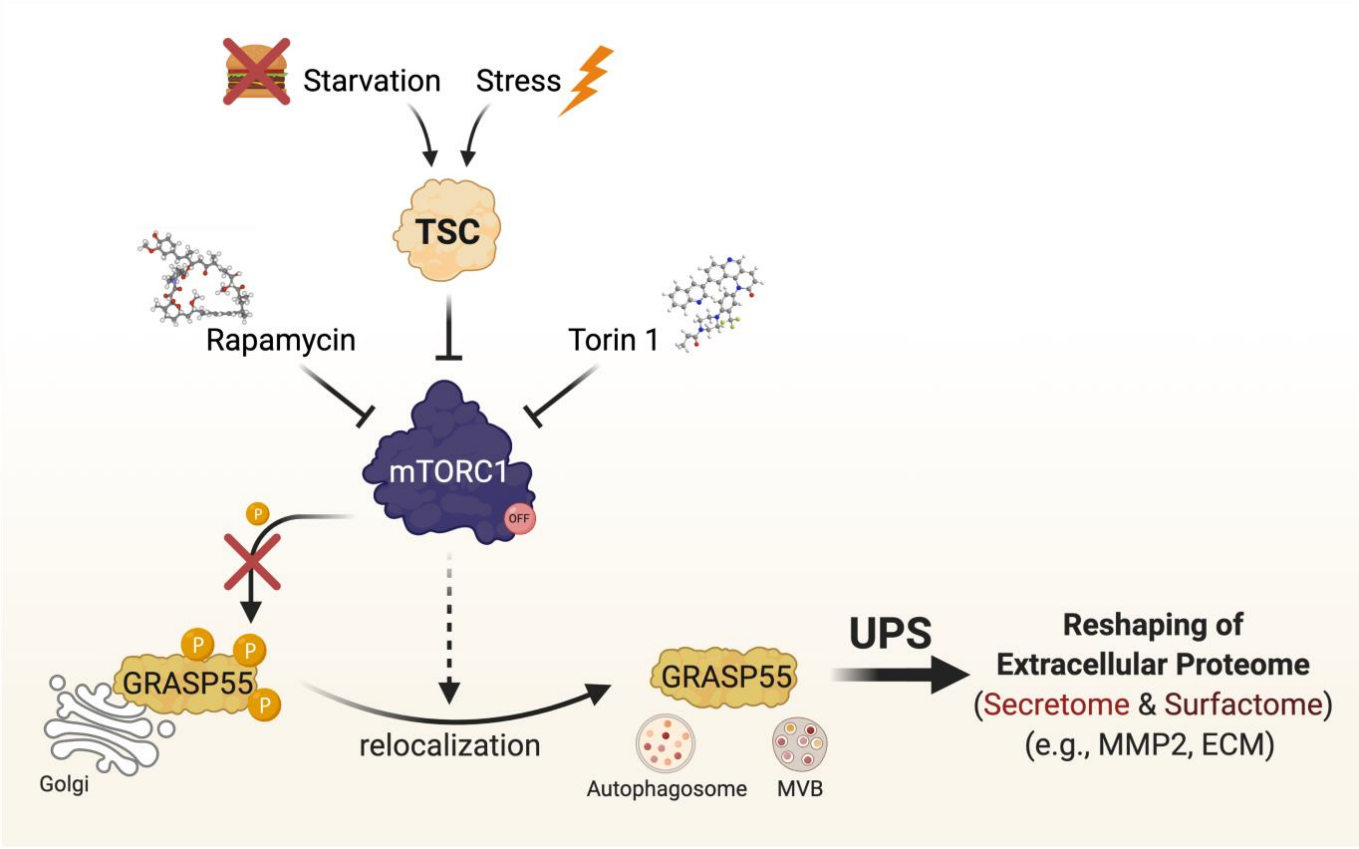
FIGURE 1: mTORC1 activity and GRASP55 relocalization control unconventional protein secretion. Active mTORC1 directly phosphorylates GRASP55 to retain it at the Golgi and prevent UPS. When cells undergo starvation, stress, or upon pharmacological mTORC1 inhibition (e.g., with Rapamycin or Torin1), dephosphorylation of GRASP55 drives its relocalization from the Golgi to vesicles of UPS, like autophagosomes and MVBs, to promote UPS of selected cargoes and to reshape the extracellular proteome (secretome and surfactome).

Only a handful of cargo proteins that use UPS to reach the cell surface or the extracellular space were previously identified. Through advanced unbiased proteomic methods, we have recently identified the GRASP55-dependent secretome (secreted proteome) and surfactome (surface proteome), thereby revealing how GRASP55 reshapes the extracellular proteome upon stress. Moreover, using an APEX2-based proximity biotinylation assay, we characterized the GRASP55 proximome/interactome at the Golgi, and showed how this is altered when mTORC1 is inhibited. This analysis, not only confirmed the aforementioned changes in GRASP55 subcellular localization, but also revealed several putative GRASP55 partners that may be contributing to the regulation of UPS or to its Golgi-related functions. In sum, these proteomics-based approaches vastly expand our understanding of UPS and will be a valuable resource serving as the foundation for future work on this important cellular function. Finally, using MMP2, one of our top secretome hits, as a proxy for UPS, we have also demonstrated the physiological outcome of changes in mTORC1-GRASP55 signaling upon stress, showing that GRASP55 is required for both the secretion of extracellular matrix (ECM) components and of enzymes that degrade and/or modify the ECM.

The mTORC1-GRASP55 signaling hub serves as the integration point in stress signaling upstream of unconventional protein secretion and coordinates the cellular adaptation to stress. Therefore, UPS is part of the general cellular stress response and not limited to the couple of stresses that were previously shown to induce it: each individual stress stimulus that signals through the TSC/mTORC1 hub is sufficient to drive GRASP55 relocalization to secretory vesicles to promote unconventional secretion of selected cargo. These findings provided an important principle of signal integration in the cellular adaptation to stress.

In sum, we recently showed how cells reshape their extracellular proteome to adapt to multiple cellular stress stimuli, highlighting the biological role of GRASP55 and UPS. In addition, we revealed the first signaling pathway that regulates UPS, thereby linking physiological stress signaling and nutrient sensing to the cellular stress response. By providing solid answers to long-standing questions about the regulation and the cell biology of UPS, we hope that our work will spark the interest of more scientists that will embark on research to better understand this important biological process. Yet, several key questions remain open: What is the exact molecular role of GRASP55 in UPS? Is it involved in cargo selection and/or sorting? Does it mediate vesicle-vesicle or vesicle-plasma membrane bridging and fusion? How and where does the selection and sorting of UPS cargo proteins happen? Given that almost half of the GRASP55-dependent secreted proteins contain a signal peptide, are the mechanisms of cargo selection different between signal-containing and signal-less proteins? What is the physiological relevance of the mTORC1/GRASP55/UPS-dependent reshaping of the extracellular proteome? We are now excited to continue our endeavor towards addressing these and other fundamental questions that will shed light on the bidirectional communication of cells with their environment.

